# Probiotic Soy Product Supplemented with Isoflavones Improves the Lipid Profile of Moderately Hypercholesterolemic Men: A Randomized Controlled Trial

**DOI:** 10.3390/nu8010052

**Published:** 2016-01-19

**Authors:** Daniela Cardoso Umbelino Cavallini, Marla Simone Jovenasso Manzoni, Raquel Bedani, Mariana Nougalli Roselino, Larissa Sbaglia Celiberto, Regina Célia Vendramini, Graciela Font de Valdez, Dulcinéia Saes Parra Abdalla, Roseli Aparecida Pinto, Daniella Rosetto, Sandro Roberto Valentini, Elizeu Antonio Rossi

**Affiliations:** 1 Departamento de Alimentos e Nutrição, Faculdade de Ciências Farmacêuticas, UNESP-Univ Estadual Paulista, Araraquara–SP 14801-902, Brasil; mamanzoni@hotmail.com (M.S.J.M.); mari_roselino@yahoo.com.br (M.N.R.); larissasbaglia@gmail.com (L.S.C.); rosely@fcfar.unesp.br (R.A.P.); rossiea@fcfar.unesp.br (E.A.R.); 2 Department of Biochemical and Pharmaceutical Technology, School of Pharmaceutical Sciences, University of São Paulo, São Paulo 05508900, Brazil; raquelbedani@yahoo.com.br; 3 Departamento de Análises Clínicas, Faculdade de Ciências Farmacêuticas, UNESP-Univ Estadual Paulista, Araraquara–SP 14801-902, Brasil; vendrasp@fcfar.unesp.br; 4 Reference Center for Lactobacilli, CERELA, S.M. Tucuman T4000ILC, Argentina; gfont@cerela.org.ar; 5 Department of Clinical and Toxicological Analyses, School of Pharmaceutical Sciences, University of Sao Paulo, São Paulo 05508900, Brazil; dspabdalla@gmail.com; 6 Departamento de Ciências Biológicas, Faculdade de Ciências Farmacêuticas, UNESP -Univ Estadual Paulista, Araraquara–SP 14801-902, Brasil; daniella.rossetto@gmail.com (D.R.); valentsr@fcfar.unesp.br (S.R.V.)

**Keywords:** fermented soy product, probiotic, isoflavones, *Enterococcus faecium* CRL183, lipid profile

## Abstract

Background: Cardiovascular disease is the leading cause of worldwide morbidity and mortality. Several studies have demonstrated that specific probiotics affect the host’s metabolism and may influence the cardiovascular disease risk. Objectives: The aim of this study was to investigate the influence of an isoflavone-supplemented soy product fermented with *Enterococcus faecium* CRL 183 and *Lactobacillus helveticus* 416 on cardiovascular risk markers in moderately hypercholesterolemic subjects. Design: Randomized placebo-controlled double-blind trial *Setting:* São Paulo State University in Araraquara, SP, Brazil. Participants: 49 male healthy men with total cholesterol (TC) >5.17 mmol/L and <6.21 mmol/L Intervention: The volunteers have consumed 200 mL of the probiotic soy product (group SP-10^10^ CFU/day), isoflavone-supplemented probiotic soy product (group ISP–probiotic plus 50 mg of total isoflavones/100 g) or unfermented soy product (group USP-placebo) for 42 days in a randomized, double-blind study. Main outcome measures: Lipid profile and additional cardiovascular biomarkers were analyzed on days 0, 30 and 42. Urine samples (24 h) were collected at baseline and at the end of the experiment so as to determine the isoflavones profile. Results: After 42 days, the ISP consumption led to improved total cholesterol, non-HDL-C (LDL + IDL + VLDL cholesterol fractions) and electronegative LDL concentrations (reduction of 13.8%, 14.7% and 24.2%, respectively, *p* < 0.05). The ISP and SP have prevented the reduction of HDL-C level after 42 days. The C-reactive protein and fibrinogen levels were not improved. The equol production by the ISP group subjects was inversely correlated with electronegative LDL concentration. Conclusions: The results suggest that a regular consumption of this probiotic soy product, supplemented with isoflavones, could contribute to reducing the risk of cardiovascular diseases in moderately hypercholesterolemic men, through the an improvement in lipid profile and antioxidant properties.

## 1. Introduction

Cardiovascular disease (CVD) is the leading cause of mortality around the world and dyslipidaemic patients remain at high risk of vascular incidents [1]. Oxidative alterations on low-density lipoproteins (LDL), resulting in oxidized LDL and electronegative LDL (LDL (‒)), are also related to atherogenic processes stimulated by the immune system components [2,3]. LDL (‒) is a modified LDL which was developed in response to its exposure to oxidizing agents (superoxide anion, hydrogen peroxide, and enzymes), which is increased in patients with hypercholesterolemia and coronary artery disease [4]. In the bloodstream, it exhibits immunogenic properties leading to the generation of autoantibodies against LDL (‒) [5]. However, the importance of anti-LDL (‒) autoantibodies in atherogenesis is controversial: the atherogenic role was supported by studies that have found elevated concentrations of autoantibody against LDL (‒) in patients suffering from atherosclerosis [6]; antiatherogenic properties were evidenced by others that have found an inverse relation between anti-LDL (‒) autoantibodies and atherosclerosis development [7,8]. Fibrinogen interferes in the blood viscosity, platelet aggregation, fibrin formation, and thus in coagulation and fibrinolysis processes. Moreover, fibrinogen is an acute phase protein involved in a systemic response to inflammation, and thus participating directly in the atherogenesis [9]. C-reactive protein (CRP) is an acute phase protein whose concentration is increased in inflammatory states at the initial stages of atherosclerotic plaque formation, and could predict future risk of sudden cardiac death in apparently healthy individuals [10].

Prevention and management of hyperlipidaemia involve pharmacological treatment and changes in the individual’s lifestyle, including practice of regular physical activity and specific diets [11]. Drugs used in the treatment of dyslipidemia are rather expensive and can produce severe side effects, which might lead to therapy discontinuation [12]. Therefore, it is important to seek new alternative strategies to modulate the lipid profile and reduce the CVD risk.

Strong evidences have suggested that the dietary intake of functional foods, such as soybean derivatives and probiotics, could positively modulate blood lipid levels [13,14,15]. Soy protein and isoflavones have been associated with the decrease of cholesterol and cardioprotective effects through anti-inflammatory and hormonal mechanisms [16,17,18]. In addition, a few studies have reported that the protective effect of soy isoflavones is limited or more pronounced in equol-producing individuals [19]. On the other hand, functional foods containing probiotics have also emerged as a potential dietary way of reducing plasma cholesterol levels [14,20]. Our research group previously showed that *Enterococcus faecium* CRL 183 is able to reduce cholesterol by 53.85% in an *in vitro* study [21]. In further studies it was demonstrated that a soy drink, fermented with *E. faecium* CRL 183, significantly improves the lipid profile in animal and clinical trials [22,23]. However, during the processing of soy beverages the total isoflavone content is drastically reduced [24]. In order to minimize this problem, the fermented soy product was supplemented with an isoflavone mixture (Isoflavin^®^, Galena: 4.7% genistin, 11.3% genistein, 5.5% daidzin, 17.8% daidzein, 2.0% glycitin and 1.0% glycitein) to obtain approximately the same amount of content of in the whole bean. This new fermented product was able to reduce the blood’s lipid levels, inhibit the atherosclerosis development and modulate the fecal microbiota of rabbits with induced hypercholesterolemia [25,26,27].

Earlier studies of our research group suggest that the daily consumption of soy drink fermented with *E. faecium* CRL 183 with isoflavones may reduce the CVD risk in animal models. Therefore, it was hypothesized that the antioxidant properties of isoflavones would be able to enhance the beneficial effect of the probiotic fermented soy product in clinical trials. In this context, the aim of the present study was to investigate the influence of an isoflavone-supplemented soy product, fermented with *E. faecium* CRL 183 and *Lactobacillus helveticus* 416 on the lipid profile and other biomarkers of CVD risk in moderately hypercholesterolemic subjects.

## 2. Experimental Section

### 2.1. Subjects and Study Design

Sixty-nine men, aged between 37 and 57 years, were initially recruited from the School of Pharmaceutical Sciences, Sao Paulo State University, Araraquara, SP, Brazil, with permission of the local ethics committee (Committee of Ethics in Research of the School of Pharmaceutical Sciences, Sao Paulo State University—approval protocol n.062007). The Local Committee follows the standards of the National Research Ethics Commission, linked to the Ministry of Health and, the study was conducted according to the guidelines laid down in the Declaration of Helsinki. A written informed consent was obtained from all subjects. The sample size was calculated using the t test for independent samples (Biostat 4.0). The data were obtained from a pilot study which was previously conducted by our research group. The primary outcome measure was change in the serum’s total cholesterol. By assuming that α = 0.05 and 1-β = 0.8, a minimum of 15 participants was required. The inclusion criteria were: men with total cholesterol (TC) >5.17 mmol/L and <6.21 mmol/L, and going on a traditional normocaloric diet (<30% of total energy as fat, 15%–18% as protein, 52%–55% as carbohydrates). The exclusion criteria were: history of chronic health problems (kidney, liver, cardiovascular, immunodeficiency or inflammatory bowel disease), consumption of any kinds of supplements, current use of hypolipemiant medication; utilization of probiotic products or antibiotic medication within the previous 3 months, and vegan dietary or soy and its derivatives consumption as a primary protein source. In this study, only male volunteers were recruited so as to exclude the natural protective effect of endogenous 17-β-estradiol (human female hormone). Asian descendants men were not allowed due to possible ethnic-specific health effects of isoflavones. Twelve participants were excluded because they did not meet the study’s inclusion/exclusion criteria and eight dropped out of the study.

### 2.2. Experimental Design and Diets

A placebo-controlled double-blind was designed and the 49 volunteers were randomly divided into 3 groups: Group ISP (*n* = 17) which received the fermented soy product with isoflavones, Group SP (*n* = 17) with the fermented soy product, and Group USP with the unfermented soy product (placebo; *n* = 15). Randomization was obtained by computer software and the products were coded with a three-digit number by an independent laboratory technician. The treatment and placebo product were packaged likewise and with the same appearance. Neither the investigators, nor the volunteers were informed about the identity of the studied products. Each volunteer consumed 200 mL of their respective drinks for 42 days. During the study, the subjects were encouraged to lead their lives as they normally would, with no change in their regular diets or physical activity. Dietary intake was recorded for three non-consecutive days to assess their habitual diet.

The fermented soy product was manufactured at UNIVERSOJA (Production and Development Unit for Soybean Derivatives) in the Food and Nutrition Department of the School of Pharmaceutical Sciences, UNESP at Araraquara (SP, Brazil), following the method described by Rossi *et al.* [28]. The chemical composition of each product is presented in Table 1. Soymilk (82.0%), soy oil (2.6%) and lactose (2.0%) were homogenized and heated to 70 °C. Following this procedure, sucrose (10.0%), skim milk (3.5%) and gelatin (0.5%) were added, and the mixture was afterwards homogenized and pasteurized (95 °C for 5 min). The fermentation (*E. faecium* CRL183 and *L. helveticus* 416; 3% *v/v*) was conducted at 37 °C until the product reached a pH of 4.4–4.5. The products were stored at 7 °C and all analyzes were conducted after 7 days. 

Only *E. faecium* CRL183 was previously confirmed as probiotic in *in vitro* and *in vivo* studies. *L. helveticus* 416 was used due to its technological properties (fermentation process improvement).

**Table 1:** Composition of the different soy-based products.

Composition in 100 g of Soy Product	ISP	SP	USP
Protein (g)	3.85 ± 0.00 ^a^	3.90 ± 0.00 ^a^	3.85 ± 0.04 ^a^
Fat (g)	2.26 ± 0.09 ^a^	2.30 ± 0.08 ^a^	2.32 ± 0.02 ^a^
Carbohydrate (g)	10.06 ± 0.11 ^a^	9.70 ± 0.15 ^a^	9.93 ± 0.16 ^a^
Ash (g)	0.90 ± 0.00 ^a^	0.90 ± 0.07 ^a^	0.90 ± 0.00 ^a^
Moisture (g)	82.93 ± 0.17 ^a^	83.20 ± 0.00 ^a^	83.00 ± 0.16 ^a^
Isoflavones (mg)	51.26 ± 1.12 ^a^	8.04 ± 0.01 ^b^	8.03 ± 0.07 ^b^

ISP = isoflavone-supplemented soy product; SP = soy product; USP = unfermented soy product (placebo). Values represent mean ± SD (*n* = 3). Statistical comparison of products (ISP, SP, USP) by ANOVA followed by a Tukey’s post hoc test. Means with identical lowercase superscript letters (a or b) in the same line do not differ significantly from each other (*p* < 0.05).

Isoflavin^®^ (Galena, Brazil) was added to the soy product before the fermentation, so as to reach 50 mg (total isoflavones) per 100 g, in order to yield the isoflavone-supplemented soy product. Isoflavin^®^ contains 40.77% total isoflavones, which consist of 1.81% genistin, 5.36% genistein, 2.91% daidzin, and 29.38% daidzein. Thus, it was added 122.64 mg of Isoflavin^®^, corresponding to: 2.22 mg of genistin, 6.57 mg of genistein, 3.57 mg of daidzin, and 36.03 mg of daidzein per 100 g of fermented product.

The placebo (non-fermented soy product) was prepared by chemical acidification (with food-grade lactic acid) of the soy product’s basic mixture (without bacterial culture or isoflavones).

Both the soy product and the isoflavone-supplemented soy product exhibited probiotic microorganism counts between 10^8^ and 10^9^ CFU (colony-forming unit)/mL.

The products were freshly produced every week, refrigerated and delivered to each volunteer in plastic vials labeled with their manufacture and expiration date.

### 2.3. Data Collection

The subject’s body mass index (BMI) was calculated using the following equation: BMI = weight (kg)/height (m^2^). Anthropometric data was gathered by using standard techniques.

Blood samples were collected after an overnight fast (12 h) at baseline (T0), after 30 (T30) and 42 (T42) days of the study. For measuring plasma LDL(‒) and anti-LDL(‒) autoantibody concentrations, the blood was drawn into evacuated tubes (Becton Dickinson, Rutherford, NJ, USA) containing EDTA (1 g/L) and centrifuged for 10 min at 3500× *g* at 4 °C (Sorvall, Kendro Laboratories Products, Ashville, USA). After the plasma separation, 1.0 mmol/L phenylmethylsulfonyl fluoride (Sigma Chemical, St Louis, MO, USA), 2.0 mmol/L benzamidine (Sigma Chemical), 2.0 mg/L aprotinin (Sigma Chemical), and 20.0 mmol/L BHT (Sigma Chemical) were added to the samples. For fibrinogen determination, the plasma was collected in tubes containing sodium citrate (38 g/L) and centrifuged at 1500× g for 15 min at 4 °C. Blood for lipid profile and C-reactive protein determination was collected using evacuated tubes, kept at room temperature and centrifuged at 3500× *g* for 10 min. One 24-h urine sample was collected at baseline and at the end of the experiment (T42) to analyze its isoflavone profile. Fecal samples were collected at days 0, 30 and 42 of the study to determine the gastrointestinal resistance of the probiotic strain. All serum, plasma, feces and urine samples were stored at −80 °C until the analysis.

### 2.4. Lipid Profile

The serum levels of TC, high-density lipoprotein cholesterol (HDL-C) and triglycerides (TG) were assayed in each volunteer, with the aid of specific enzyme kits. The TC was measured by the cholesterol fast color method [29]. The HDL cholesterol was estimated by selective precipitation of lipoproteins [30] and then by TC method in the supernatant. The triglyceride fast color method was used to measure the triglycerides [31]. The LDL-C (low-density lipoprotein cholesterol) was determined by Friedewald’s equation LDL-C = TC − HDL-C − TG/5 [32]. The Non-HDL cholesterol was calculated by subtracting HDL-C from TC, representing the LDL + IDL + VLDL cholesterol fractions [33,34]. The concentrations of all lipids parameters were expressed in mmol/L.

### 2.5. LDL (‒) and Anti-LDL (‒) Autoantibodies

The LDL fraction was separated by ultracentrifugation as described by Lobo *et al.* [35]. Concentrations of LDL (‒) were detected in the blood plasma by ELISA using two anti-electronegative LDL monoclonal antibodies (mAb 1A3 and mAb 2C7), according to Faulin *et al.* [36]. Briefly, ninety-six-well flat-bottom polystyrene plates (Costar, Corning, Inc., Teterboro, NY, USA) were coated with 50 µL mAb 1A3 (1 µg/well) in a carbonate-bicarbonate buffer (pH 9.4, 0.1 M) and incubated overnight at 4 °C. Subsequently, each microplate was washed three times with a phosphate-buffered saline solution (PBS; Tris-HCl 50 mM and NaCl 150 mM, pH 7.4) containing Tween 20 (0.5%) and blocked with 5% nonfat dry milk for 2 h at 37 °C. The microplates were then washed once more and incubated with 50 µL plasma for 2 h at 37 °C. The plates were washed and incubated with 2C7 anti-LDL monoclonal antibody biotinylated for 2 h at 37 °C. After washing, the microplates were incubated with a streptavidin- HRP (horseradish peroxidase) conjugate (Rockland Immuno- chemicals for Research, Gilbertville, PA, USA) for 1 h at 37 °C. Then, the OPD substrate solution was added to each well. The absorbance intensity was determined immediately using a microplate reader (SpectraCount Microplate Photometer, Packard Instruments Company, Downers Grove, IL, USA). The calibration curve was made with the LDL obtained from human plasma. All samples and standards were run in triplicate. The intra and inter-assay variations for the ELISA test were 8% and 15%, respectively.

Anti-LDL (‒) autoantibodies were determined according to Faulin *et al.* [29]. Ninety-six-well flat bottom polystyrene plates were coated with 1 μg/mL of LDL(−), overnight at 4 °C. The plates were washed 3 times with PBS, pH 7.4, containing 0.05% Tween 20. The plates were blocked by the addition of PBS containing 2% non-fat dry milk and 0.01% Tween 20 for 90 minutes at 37 °C, followed by washing, as stated above. Plasma in PBS containing 1% non-fat milk and 0.01% Tween 20 was added in the plates and incubated for 1 1/2 h at 37 °C. Afterwards, the plates were washed and incubated with anti-human IgG-conjugated to horseradish peroxidase (Bio-Rad) for 1 h at 37 °C. Then, the washed plates were incubated with 3, 3′, 5, 5′ tetramethylbenzidine (TMB, Sigma Chemical) for 10 min at 37 °C. The reaction was interrupted by adding 0.5 mol/L sulfuric acid, and the absorbance at 450 nm was measured by spectrophotometry, using a plate reader (Synergy™ Mx, Biotek Instruments Inc, Winooski, VT, USA).

### 2.6. C-Reactive Protein (CRP) and Fibrinogen

The concentration of fibrinogen was assayed by the Gauss method, using a commercial kit (Wiener Laboratorios A.S.I.C., Rosario, Argentina).

The C-reactive protein was analyzed in the Clinical Laboratory of the University Hospital at the University of Sao Paulo (USP) using the BN II Systems (Siemens) by immunonephelometry method, with detection limit of 1.43 nmol/L (0.15 mg/L) according to manufacturer’s instructions.

### 2.7. Isoflavone Profile

The extraction of isoflavones, including daidzin, genistin, daidzein, genistein, and equol, was performed according to Rossi *et al.* [24]. Isoflavone standards were purchased from Sigma Chemicals Co. (St. Louis, MO, USA) and prepared in high performance liquid chromatography (HPLC) grade ethanol. The isoflavone quantification was carried out through a HPLC system (Shimadzu^®^, Kyoto, Japan), which is equipped with an auto sampler (SIL-10AF), a diode array ultraviolet (UV) visible detector (SPD-10MA), a quaternary pump, a vacuum degasser, and a Hypersil ODS C18 (250 mm × 4.6 mm) reverse-phase column (Supelco^®^). All reagents used in the isoflavone extraction and HPLC analyses were filtered through a 0.22 μm or 0.45 μm membrane (hydrophilic Polytetrafluoroethylene—PTFE—Millipore). The HPLC isocratic elution was used to isolate the isoflavones for detection and was composed of acetic acid-water 60% (2:98 *v/v*—solvent A) and methanol-acetonitrile 40% (80:20 *v/v*—solvent B), set at a flow rate of 1.1 mL/min, during 40 min. The diode array UV-visible detector was set at dual wavelengths of 262 nm to detect daidzin, genistin, daidzein and genistein and 280 nm for equol detection. The identification of isoflavones was confirmed by the HPLC retention time and UV spectral analysis.

### 2.8. Gastrointestinal Resistance of E. faecium

The fecal samples (1.0 g) were homogenized, suspended in peptone water (9.0 mL) and serially diluted. *Enterococcus* spp. colonies were isolated using the KF *Streptococcus* agar (Acumedia, Baltimore, MD, USA) with incubation at 37 °C for 48 h. Colonies with distinct morphologies were picked out and transferred onto the Bile Esculin Azide agar for confirming of the *Enterococcus* genus (Acumedia) [37,38,39]. The API Strep 20 (Biomérieux, Marcy l’Etoile, France) was used to identify the enterococci species. Colonies identified as *E. faecium* were cultured on Tryptic Soy Broth (TSB, Acumedia) for genomic DNA extraction. *Enterococcus faecium* species confirmation was performed by Polymerase Chain Reaction (PCR) amplification of genomic DNA. The genomic DNA was extracted by using a DNeasy kit (Qiagen, Valencia, CA, USA), according to manufacturer’s instructions. A pure culture of *E. faecium* CRL 183 was included for comparison. 16S rDNA PCR amplification was performed using the following primer combination: Enf 1 (1 (5′-ATTACGGAGACTACACACTTTG-3′) and Ent 2 (5′- TAGCCATAGAAGTTACATCAAG-3′). These primers encode the 16S rRNA region of *E. faecium*. The reaction mixture was composed of 1 μM of each primer, 100 ng of genomic DNA, 1.5 mmol/L MgCl2, 100 μmol of deoxynucleotides and 2 U of Taq DNA polymerase enzyme (final volume was adjusted to 50 μL). The mixture was amplified using the following program: 30 cycles at 94 °C for 1 min, 56 °C for 30 s and 72 °C for 1 min and a final extension at 72 °C for 5 min. PCR products were separated by electrophoresis in agarose gel (1.5% in TAE buffer—40 nmol/L Tris, 11% glacial acetic acid, 1 mmol/L EDTA). A 1 kb Ladder Plus (Invitrogen, Life Technologies, Carlsbad, CA, USA) was included as molecular size standard. Finally, the gels were stained with ethidium bromide and photographed under UV illumination [39,40].

### 2.9. Statistical Analysis

Quantitative results were reported as mean ± SD. Normality and homogeneity were checked before conducting further analyses. Parametric variables were analyzed by univariate analysis of variance (ANOVA) followed by a Tukey’s post hoc test. The relation between the individual’s ability to produce equol and the cardiovascular risk markers was analyzed by Pearson’s correlation test. Statistical significance was declared when *p* < 0.05. All analyses were carried out with the BIOSTAT statistical package.

## 3. Results

### 3.1. Study Population

The study groups’ age ranged between around 45 and 48 years, with BMI between 25 and 26 kg/m^2^ and waist-to-hip circumference ratio of around 0.90. At baseline, no significant difference in anthropometric characteristics and food intake was observed between the experimental groups (*p* < 0.05). The overall clinical status of the volunteers was satisfactory, two of them being hypertensive (ISP and SP groups) and two having gastrointestinal diseases (ISP and SP) (Table 2).

No significant changes (*p* < 0.05) were observed in the anthropometric measurements of the volunteers during the study period (data not shown). Clinical conditions, lifestyle and caloric intake also remained unchanged during the protocol.

**Table 2:** Clinical characteristics at baseline of the 49 men involved in the study.

Clinical Characteristics	Groups		
	ISP (*n* = 17)	SP (*n* = 17)	USP (*n* = 15)
Age (y)	48.1 ± 5.1 ^a^	46.1 ± 6.1 ^a^	45.4 ± 5.1 ^a^
Height (m)	1.73 ± 0.08 ^a^	1.75 ± 0.06 ^a^	1.72 ± 0.07 ^a^
Weight (kg)	76.7 ± 14.5 ^a^	81.6 ± 15.6 ^a^	78.0 ± 14.1 ^a^
BMI (kg/m^2^)	25.65 ± 4.36 ^a^	26.41 ± 4.88 ^a^	26.26 ± 3.98 ^a^
WHR	0.91 ± 0.06 ^a^	0.90 ± 0.07 ^a^	0.90 ± 0.05 ^a^
***Diabetes***	0	0	0
Hypertension	1 (5.9%)	1 (5.9%)	0
Gastrointestinal diseases	1 (5.9%)	1 (5.9%)	0
Previous CVD	0	0	0
***Smoking***	3 (17.6%)	2 (11.8%)	3 (20.0%)

Values are mean ± SD. Statistical comparison of groups (ISP, SP, USP) by ANOVA followed by a Tukey’s post hoc test. Means with identical lowercase superscript letter (a) in the same line do not differ significantly from each other (*p* < 0.05). ISP Group: individuals who consumed the isoflavone-supplemented soy product; SP Group: individuals who consumed the soy product; USP Group: individuals who consumed the unfermented soy product (placebo). BMI = Body mass index; WHR = waist–hip ratio; CVD = cardiovascular disease. Normal values: BMI > 18.5 and <25 (kg/m^2^); WHR < 0.90 cm [41].

### 3.2. Lipid Profile

Only the subjects who consumed ISP showed a significant reduction of 13.8% ± 7.7% on the basal levels of TC, throughout 42 days of study. Serum levels of HDL-C and triglycerides did not differ significantly between groups in this study. At the end of the experimental protocol, there was a reduction of 24.6% ± 8.6% (*p* < 0.05), 11.0% ± 4.8% (*p* = 0.171) and 8.1% ± 3.1% (*p* = 0.338) in the HDL-C levels of groups USP, ISP and SP, respectively (Table 3). After 30 and 42 days of treatment, ISP intake resulted in a decrease in the LDL-C (14.8% ± 9.7% and 13.5% ± 8.7%, respectively) and non-HDL-C (15.5% ± 11.2% and 14.7% ± 10.3%, respectively) concentrations, but this effect was significant only in comparison with the USP group (*p* < 0.05). At the end of the study, the TC-to-HDL-C ratio was lower (*p* < 0.05) in the ISP group if compared to the USP group.

**Table 3:** Serum lipids levels during the experiment.

Time	ISP (*n* = 17)	SP (*n* = 17)	USP (*n* = 15)
TC (mmol/L)			
T0	5.47 ± 0.59 ^a,A^	5.76 ± 1.02 ^a,A^	5.60 ± 0.76 ^a,A^
T30	4.65 ± 0.48 ^b,B^	5.44 ± 0.93 ^a,A^	5.52 ± 0.76 ^a,A^
T42	4.72 ± 0.45 ^b,B^	5.41 ± 0.86 ^a,A^	5.50 ± 0.78 ^a,A^
HDL-C (mmol/L)			
T0	1.36 ± 0.22 ^a,A^	1.38 ± 0.25 ^a,A^	1.50 ± 0.28 ^a,A^
T30	1.18 ± 0.23 ^a,A^	1.33 ± 0.19 ^a,A^	1.24 ± 0.38 ^a,A,B^
T42	1.21 ± 0.22 ^a,A^	1.28 ± 0.18 ^a,A^	1.13 ± 0.38 ^a,B^
LDL-C (mmol/L)			
T0	3.23 ± 0.61 ^a,A^	3.54 ± 0.92 ^a,A^	3.14 ± 0.95 ^a,A^
T30	2.75± 0.51 ^a,A^	3.33 ± 0.84 ^a,A^	3.41 ± 0.60 ^a,A^
T42	2.79 ± 0.50 ^b,A^	3.35 ± 0.80 ^a,b,A^	3.51 ± 0.63 ^a,A^
TG (mmol/L)			
T0	1.91 ± 0.98 ^a,A^	1.88 ± 0.88 ^a,A^	1.97 ± 1.32 ^a,A^
T30	1.57 ± 0.74 ^a,A^	1.83 ± 0.89 ^a,A^	2.18 ± 1.61 ^a,A^
T42	1.55 ± 0.70 ^a,A^	1.86 ± 0.93 ^a,A^	2.15 ± 1.64 ^a,A^
nHDL-C (mmol/L)			
T0	4.11 ± 0.65 ^a,A^	4.38 ± 1.12 ^a,A^	4.08 ± 0.82 ^a,A^
T30	3.47 ± 0.48 ^a,B^	4.11 ± 0.98 ^a,A^	4.28 ± 0.99 ^a,A^
T42	3.50 ± 0.46 ^b,B^	4.14 ± 0.92 ^a,b,A^	4.37 ± 0.96 ^a,A^
TC/HDL-C			
T0	4.11 ± 0.78 ^a,A^	4.28 ± 0.90 ^a,A^	3.80 ± 0.83 ^a,A^
T30	4.07 ± 0.75 ^a,A^	4.18 ± 0.90 ^a,A^	4.92 ± 1.69 ^a,A^
T42	4.00 ± 0.71 ^b,A^	4.32 ± 0.83 ^a,b,A^	5.47 ± 1.98 ^a,A^

Values are mean ± SD. ANOVA followed by a Tukey’s post hoc test. Statistical comparison of groups (ISP, SP, USP): means with identical lowercase superscript letters (a or b) in the same line do not differ significantly (*p* < 0.05) from each other, in the same period. Statistical comparison of periods (T0, T30, T42): means with identical uppercase superscript letters (A or B) in the same column do not differ significantly from each other (*p* < 0.05), for the same parameter. TC: total cholesterol; LDL-C: low-density lipoprotein; HDL-C: high-density lipoprotein; TC/HDL-C: total cholesterol to HDL-cholesterol ratio; TG: triglyceride; nHDL-C: non HDL-cholesterol. ISP Group: individuals who consumed the isoflavone-supplemented soy product; SP Group: individuals who consumed the soy product; USP Group: individuals who consumed the unfermented soy product (placebo).T0: baseline; T30: 30 days of daily consumption of soy products; T42: 42 days of daily consumption of soy products. Normal values for subjects at low or moderate risk: CT: <5.0 mmol/L; HDL-C: >1.6 mmol/L (ideal); LDL-C: <3.0 mmol/L; TG: <1.7 mmol/L [41].

### 3.3. LDL (‒) and Anti-LDL (‒) Autoantibodies

**Figure 1:**
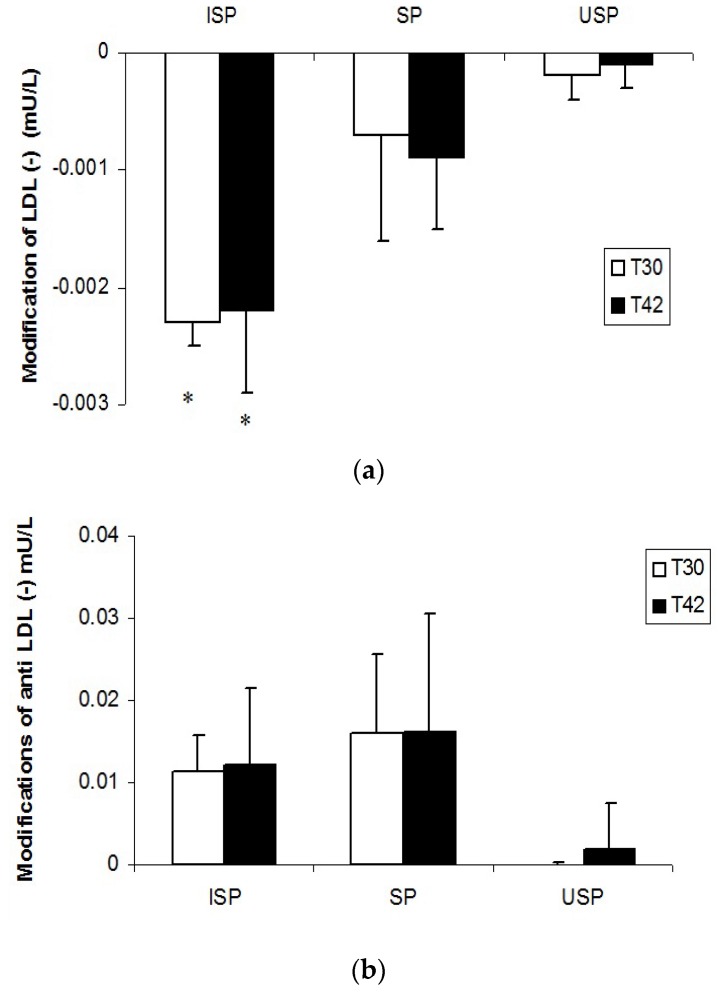
Electronegative low-density lipoproteins (LDL) (LDL (‒)) (**a**) and anti-LDL (‒) autoantibodies (**b**) after 30 and 42 days of study (changes from baseline). * Different from the baseline (*p* < 0.05). ISP Group: individuals who consumed the isoflavone-supplemented soy product; SP Group: individuals who consumed the soy product; USP Group: individuals who consumed the unfermented soy product (placebo). T0: baseline; T30: 30 days of daily consumption of soy products; T42: 42 days of daily consumption of soy products.

LDL(‒) levels in the ISP group were reduced after 30 (25.3% ± 9.9%) and 42 days (24.2% ± 11.1%) of study in comparison with the baseline (*p* < 0.05). After 30 and 42 days of study, the concentration of autoantibody against LDL (‒) tended to be higher in subjects who consumed the fermented product supplemented or not with isoflavones (Figure 1).

### 3.4. Fibrinogen and C-Reactive Protein

**Figure 2:**
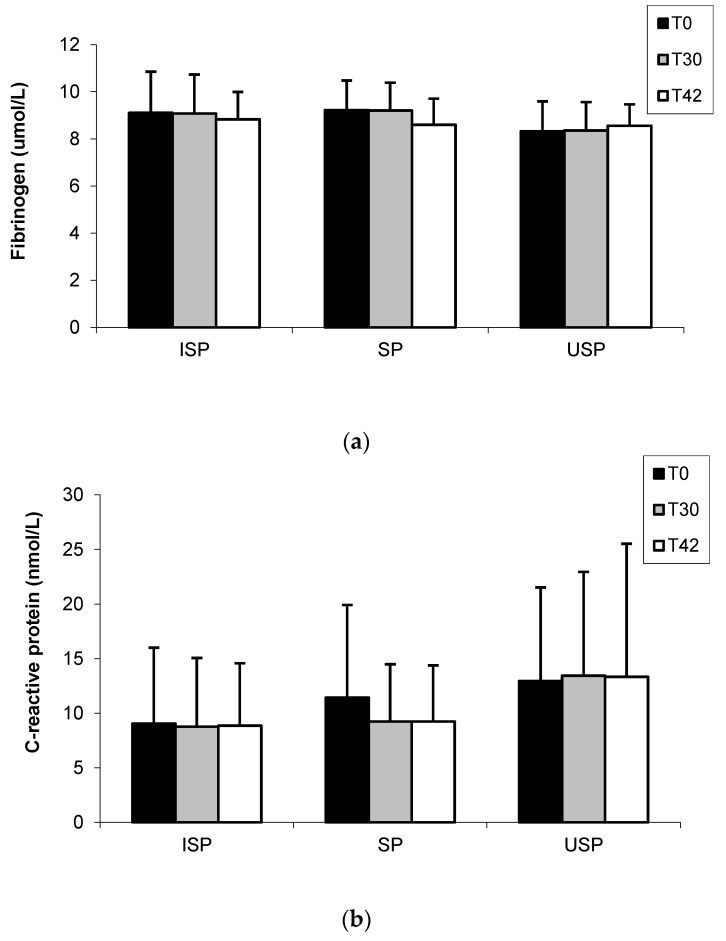
Fibrinogen (**a**) and C-reactive protein (**b**) concentrations during clinical trial. Values are mean ± SD. Means without significant differences between groups for the same sampling period of the study and without significant differences between sampling periods for the same study group (*p* < 0.05). ISP Group: individuals who consumed the isoflavone-supplemented soy product; SP Group: individuals who consumed the soy product; USP Group: individuals who consumed the unfermented soy product (placebo). T0: baseline; T30: 30 days of daily consumption of soy products; T42: 42 days of daily consumption of soy products.

At the end of the study (T42), there was a reduction in the fibrinogen concentration of volunteers who received the SP (T0: 9.22 ± 1.25; T42: 8.60 ± 1.10 μmol/L) and ISP (T0: 9.10 ± 1.77; T42: 8.83 ± 1.16 μmol/L), respectively. On the other hand, the group that ingested USP exhibited increased levels of fibrinogen (T0: 8.32 ± 1.27; T42: 8.56 ± 0.90 μmol/L). However, these results were not statistically significant (*p* < 0.05) (Figure 2a). The C-reactive protein levels of ISP (T0: 9.05 ± 6.955; T42: 8.86 ± 6.38 nmol/L) SP (T0: 11.43 ± 8.48; T42: 9.24 ± 5.14 nmol/L) and USP (T0: 12.95 ± 8.57; T42: 13.33 ± 12.19 nmol/L) groups did not differ (*p* < 0.05) during the experimental period (Figure 2b).

### 3.5. Urine Isoflavones

At the end of the experimental protocol, subjects who ingested ISP showed a total isoflavone level that was 5.09 and 7.50 times greater than that of those who consumed the SP and USP, respectively (Table 4). The volunteers were defined as “equol producers” when the log10-trzansformed urinary S-equol: daidzein ratio was greater than −1.75 [42]. Thus, equol was only identified in the ISP group, and 66.7% of volunteers were classified as “equol producers”. There was no significant correlation between individual ability to produce equol and the cardiovascular risk markers analyzed in this study (Pearson’s correlation, *p* < 0.05: CT = −0.0668; HDL-C= −0.0426; LDL-C= 0.4636; autoantibody against LDL (‒) = −0.3831; CRP = 0.3149; and fibrinogen = 0.4754), except for LDL (‒) = −0.6938.

**Table 4:** Urine isoflavone profile of volunteers from different groups at the end of study (42 days).

Isoflavones (μmol/L)	ISP (*n* = 17)	SP (*n* = 17)	USP (*n* = 15)
Daidzein	70.09 ± 25.21 ^a^	13.02 ± 0.67 ^b^	6.37 ± 1.9 ^c^
Equol	5.24 ± 2.68	nd	nd
Genistein	23.42 ± 7.88 ^a^	6.18 ± 0.11 ^b^	6.55 ± 0.41 ^b^
Total	75.75 ± 35.78 ^a^	19.20 ± 0.79 ^b^	12.92 ± 0.60 ^c^

Values are mean ± SD. Statistical comparison of groups (ISP, SP, USP) by ANOVA followed by a Tukey’s post hoc test. Means with identical lowercase superscript letter (a, b or c) in the same line do not differ significantly from each other (*p* < 0.05). Statistical comparison between groups: means with identical lowercase superscript letters in the same line do not differ significantly (*p* ≤ 0.05). ISP Group: individuals who consumed the isoflavone-supplemented soy product; SP Group: individuals who consumed the soy product; USP Group: individuals who consumed the unfermented soy product (placebo).

### 3.6. E. faecium Gastrointestinal Survival

In general, there was an increase in the *E. faecium* population in the ISP and SP groups (Table 5).

**Table 5:** *Enterococcus* species (%) isolated from the feces of volunteers in 0, 30 and 42 days of the experimental protocol.

Treatments	*Enterococcus* Species					
	T0		T30		T42	
ISP	*E. faecium*	48.36%	*E. faecium*	93.97%	*E. faecium*	96.09%
	*E. faecalis*	30.19%	*E. durans*	6.03%	*E. durans*	3.91%
	*E. durans*	21.45%				
SP	*E. faecium*	54.55%	*E. faecium*	83.00%	*E. faecium*	83.00%
	*E. gallinarum*	18.55%	*E. durans*	17.00%	*E. durans*	17.00%
	*E. durans*	26.90%				
USP	*E. faecium*	52.80%	*E. faecium*	50.30%	*E. faecium*	45.10%
	*E. durans*	20.07%	*E. gallinarum*	25.80%	*E. gallinarum*	30.10%
	*E. faecalis*	7.13%	*E. durans*	23.90%	*E. durans*	24.80%

ISP Group: individuals who consumed the isoflavone-supplemented soy product; SP Group: individuals who consumed the soy product; USP Group: individuals who consumed the unfermented soy product (placebo). SP: individuals who consumed soy product; Group USP: individuals who consumed unfermented soy product (placebo).

**Figure 3:**
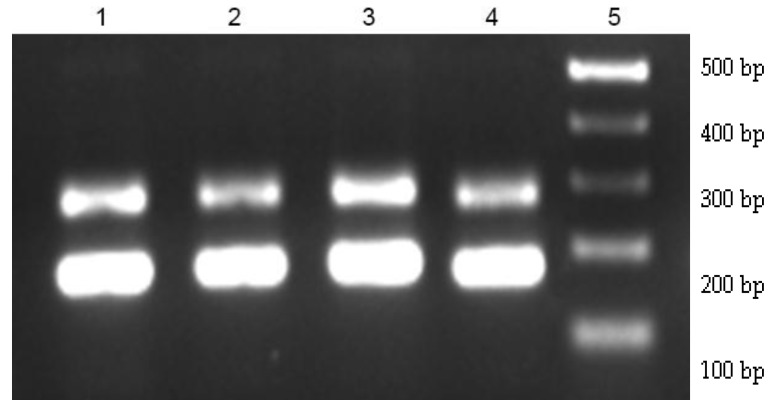
Agarose gel electrophoresis of Polymerase Chain Reaction (PCR) products obtained from colonies isolated from fecal samples after 42 days of intervention. 1—*E. faecium* from ISP; 2—*E. faecium* from SP; 3—*E. faecium* from USP; 4—*E. faecium* CRL 183 (pure strain); 5—1 kb DNA ladder.

The PCR amplification (Figure 3) indicated that, at the beginning of the study (T0), approximately 50% of *Enterococcus* spp. colonies isolated from volunteers of different groups were confirmed as belonging to the *E. faecium* species. At the end of the study, 96.09% and 83.00% of the colonies isolated from volunteers of ISP and SP groups, respectively, were confirmed as *E. faecium*. Nevertheless, only 45.10% of the colonies from the USP group were *E. faecium*. PCR products obtained from the genomic DNA of the pure culture of *E. faecium* CRL 183 indicated that species isolated from the volunteer‘s feces belong to the CRL 183 strain.

## 4. Discussion

At the beginning of the study all groups of volunteers had average levels of TC above 5.18 mmol/L, LDL-C between 3.14 and 3.54 mmol/L, HDL-C above 1.29 mmol/L and TG higher than 1.69 mmol/L. It is important to point out that no significant baseline differences were observed (*p* < 0.05) for any of the evaluated lipid parameters, which enabled a comparison between groups during the study.

After 42 days, the groups’ comparison showed that the ISP presented lower concentrations of TC (4.72 ± 0.45 mmol/L), LDL-C (2.79 ± 0.50 mmol/L), non-HDL-C (3.50 ± 0.46 mmol/L) and CT/HDL-C ratio (4.11 ± 0.78), differing significantly from the placebo group (USP) in all these parameters, and only in TC from the SP group. The fermented soy product, either being supplemented with isoflavones or not, also prevented the reduction of HDL-C during the experimental protocol.

In a previous study, Rossi *et al.* [23] found that subjects who received the same fermented product without supplementation (SP) showed unchanged TC and LDL-C concentrations and increased HDL-C levels by 10% throughout 6 weeks of intervention. The differences between the results of the previous study and those in the present investigation can be explained by the clinical characteristics of the participants, since Rossi *et al.*, evaluated normocholesterolemic men.

Several other studies have been conducted in order to verify the effects of probiotic microorganisms in blood lipids, but the results are still controversial. Naruszewicz *et al.* [43] concluded that the ingestion of a drink containing *Lactobacillus plantarum* 299v promotes a positive change (LDL-C = −12%; HDL-C = 10%) in plasma lipid levels in male smoking individuals. Hlivak *et al.* [44] reported that the consumption of *E. faecium* M-74 capsules associated with 50 μg of selenium led to a decrease in TC (12%) and LDL-C (20%). In a clinical study conducted by Ejtahed *et al.* [45], the ingestion of yoghurt (300 g), produced with *Lactobacillus acidophilus* and *Bifidobacterium lactis* was able to decrease TC and LDL-C levels. Capsules containing three strains of *L. plantarum* (CECT 7527, 7528 and 7529; 1.2 × 10^9^ CFU) also promoted a reduction of TC levels [46]. On the other hand, the administration of *Lactobacillus paracasei* LTH 2579 [47], *Lactobacillus fermentum* (PCC^®^) [48] and *Lactobacillus rhamnosus* GG (LGG) [49] did not result in a hypocholesterolemic effect. The discrepancies observed in scientific literature may be due to the used microorganism strains, the population chosen for the study (normo or hipercholesterolemic), the vehicle of probiotic administration and the protocol duration.

The mechanisms of hypolipemiant action of probiotics involves the assimilation of cholesterol, deconjugation of bile salts, fermentation of non-digestible carbohydrates from the diet producing short-chain fatty acids (SCFA), and microbiota modulation [20,50,51].

Chen *et al.* [50] evaluated the effect of *Lactobacillus rhamnosus* hsryfm 1301 or its fermented milk on the intestinal microbiota and its relation with the serum lipids profile in a hyperlipidemic rat model. The results showed that the microbiota and lipid profile were improved after probiotic intervention. A positive correlation between *Ruminococcus* spp. and triglycerides; *Dorea* spp. and TC and LDL-C; and between *Enterococcus* spp. and HDL-C was observed. On the other hand, *Butyrivibrio* spp. was negatively correlated with TC and LDL-C (*p* < 0.05), since this bacterial genus has activity of linoleate isomerase, which could decrease the mRNA expression of sterol regulatory element-binding protein (SREBP)-1c.

Oxidative modifications in LDL levels are usually related to an inflammatory process which is associated with atherosclerosis. Particles of LDL (‒) induce the expression of adhesion molecules to leukocytes, leading their infiltration in the arterial wall. Monocytes differentiate into macrophages with "scavenger" receptor properties that internalize lipid particles and promoting the ateroma formation [4,52]. LDL (‒) also induce the formation of autoantibodies reactive to LDL (−), which have an uncertain role in the formation of the atherosclerotic plaque. Lobo *et al.* [35] reported that the anti-LDL (−) IgG level were negatively correlated with inflammation marker concentration (TNF-α, ICAM-1 and VCAM-1) in hemodialysed patients. Grosso *et al.* [53] showed that the immunization with anti-LDL (−) monoclonal antibody reduced the free LDL (−) levels in the bloodstream and prevented their atherosclerotic action. This effect could be due to the formation of immune complexes to LDL (−) that neutralize the LDL (‒) particles.

In the present study, the ISP consumption significantly reduced the LDL (‒) concentrations if compared to the baseline period. A trend of elevation on the levels of anti-LDL (‒) autoantibodies was observed in the ISP and SP groups, but this effect was not significant (*p* < 0.05). It can be assumed that LDL (‒) would stimulate the production of anti-LDL (−) autoantibodies, and the posterior formation of immune complexes to LDL (‒), which could be involved in the reduction of LDL (‒) levels in the volunteers. However, the results of the present study do not allow the definition of the exact role of these markers in CVD, because all volunteers were hypercholesterolemic and had no diagnosed coronary heart disease.

The lipid-lowering potential of isoflavones has been extensively studied and the results are not uniform. Studies with hypercholesterolemic men showed a significant reduction of TC, non-HDL-C [54] and LDL-C [55] after consumption of isolated soy protein containing varying amounts of isoflavones. By analyzing 22 clinical studies, Sacks *et al.* [56] found that the combination of soy protein and isoflavones was able to reduce approximately 3% of LDL-C level. However, other studies did not demonstrate the beneficial effect of isoflavones on serum lipids. In this line, Hodgson *et al.* [57] found that the administration of 55 mg of isoflavones in tablet did not significantly alter the levels of serum lipids after 8 weeks of study. West *et al.* [58] concluded that ingestion of the 25 g of soy protein containing 90 mg of isoflavones for 6 weeks resulted in a lipid profile improvement of the volunteers.

Isoflavones can improve the lipid profile and reduce the risk of cardiovascular disease by estrogenic and non-estrogenic-related mechanisms. 17-β-estradiol enhance nitric oxide bioactivity and modulate lipoprotein levels. Estrogen–estrogen receptor complexes act as transcription factors that promote gene expression with vascular effects (response to injury and regulation of vasomotor tone) related to cardioprotective properties [59]. Considering that isoflavones are structurally similar to the 17-β-estradiol (human female hormone), they can bind to estrogen receptors (ER) and have estrogen-like activities. The cardioprotective effect of isoflavones by estrogenic and non-estrogenic-related mechanisms include: decrease in LDL cholesterol level, modulation of immune system (inhibition of pro-inflammatory cytokines, cell adhesion proteins and inducible nitric oxide production), protection of LDL oxidation, inhibition of platelet aggregation and improvement of vascular reactivity [60]. The antioxidant effect are derived from the ability of scavenge radicals, chelate metals and improvement of activities of antioxidant enzymes (superoxide dismutase, catalase and glutathione peroxidase) [61]. Isoflavones can also modulate cholesterol metabolism, promoting hepatic cholesterol catabolism [62].

The main soy isoflavones are in the glycosidic (genistin, daidzin and glycitin) or aglycone (genistein, daidzein and glycitein) form, and the latter shows diagnosed higher bioavailability and associated biological effects. Daidzein and genistein exhibit more pronounced lowering effects of cholesterol if compared to their respective glucosides forms [61]. It is noteworthy that the glycosidic isoflavones are hydrolysed in the small intestine to the aglycone form and the metabolization efficiency depends on the intestinal microbiota composition. The hypotheses to explain the differences in the results obtained with isoflavones intake include: the amount and composition of the isoflavone mixture and the individual capacity to metabolize daizein to equol, which is a metabolite that exhibits a potent antioxidant effect *in vivo* [63].

The isoflavone recovery in the urine was 24.80% ± 7.89%, 31.03% ± 0.93% and 21.11% ± 3.11% for groups ISP, SP and USP, respectively, with daidzein being eliminated at higher concentrations. The low recovery of isoflavones could be explained by their metabolites production, which was not determined in the present study. It is also important to note that the excretion of daidzein in the SP group was significantly greater than that of group USP. These results suggest that the used lactic acid bacteria (*E. faecium* CRL 183 and *L. helveticus* 416) and/or metabolites produced during the fermentation can influence the conversion of isoflavone glucosides into aglucones.

Only 20% to 50% of the adult human population is able to produce equol after ingesting pure isoflavones or soy-based foods [64,65,66,67]. The individual variation in the intestinal microbiota composition and genetic predisposition are related to the presence of equol producers or non-producers in populations with similar diets containing isoflavones [68]. In the present study, the percentage of equol producers in the group that consumed ISP was superior to that presented in scientific literature (66%) (data not shown). However, it was not possible to determine whether the individual capacity to produce equol was affected by the ingestion of the product or due to the presence of the probiotic microorganism, since at the beginning of the experiment (T0), the volunteers did not consume foods with a significant source of isoflavones and the basal levels of these substances in urine have not reached the detection limit of the method.

Wong *et al.* [69] studied the relations between equol production after regular consumption of soy foods and lipid profile modulation. The results showed that soy foods reduced LDL-C in equol producers and non-producers. However, HDL-C concentrations were higher in volunteers that are able to produce equol. A correlation between improved lipid profile and equol production was not observed in the present study. Nevertheless, equol production by subjects in the ISP group was inversely correlated with the LDL (‒) profile, indicating that the antioxidant capacity of this metabolite could reduce the susceptibility to oxidation of this lipoprotein. The mechanism related to this effect involves the inhibition of superoxide (O_2_) production that enhances the levels of free NO, which prevents LDL modification [70].

The probiotic microorganisms should reach the intestine in a viable form to promote their beneficial effects. The exposure to the gastrointestinal environment, which includes stomach acid, bile salts and enzymes, represents the main survival hurdle for the probiotic bacteria [71], being necessary to evaluate the resistance of individual strains under these conditions. In the present study, probiotic *E. faecium* CRL 183 resistance was evaluated by biochemical and molecular tests. Colonies with positive identification of *Enterococcus* genus by the API 20 Strep test were submitted to PCR using a species-specific primer combination (Enf 1 and Ent 2) to amplify the 16S rRNA gene region from the genomic DNA of *E. faecium* CRL 183, thus generating two amplification products: one of 200 bp and another of 300 bp. The observation of two bands (200 bp and 300 bp) is in agreement with the results obtained by Sivieri *et al.* [39] and Bedani *et al.* [37], who evaluated the viability of *E. faecium* CRL183 in rats that receive the probiotic pure culture or a fermented soy product, during 30 weeks and 30 days, respectively. Langa *et al.* [40], using the Enf 1—Ent 2 primers, obtained two DNA bands after amplification from the other strains of *E. faecium* (V8, HA1 and CH3): a 300-bp band and a unexpected second band of 400 bp. It is interesting to note that the *E. faecium* CRL39 amplification demonstrated the same result, while most *E. faecium* found in the feces of volunteers who consumed the test products (ISP and SP) exhibited similar PCR products obtained from the genomic DNA of the pure culture of *E. faecium* CRL 183. This result is an indicative that the probiotic microorganism used in the soy products, either supplemented or not with isoflavones, can survive to the adverse conditions of the human gastrointestinal tract, furthermore, it could also assist in the intestinal microbiota modulation, collaborating with the observed lipid-lowering effect.

## 5. Conclusions

The results of the present study suggest that the regular consumption of isoflavone-supplemented soy product fermented with *E. faecium* CRL 183 and *L. helveticus* 416 could contribute to a reduction of CVD risk markers in moderately hypercholesterolemic men, by lipid profile improvement, besides reducing the oxidation of LDL particles. The beneficial health effects observed herein probably involve a combination of antioxidant properties from isoflavones and the anti-inflammatory action of the probiotic strain. It is important to emphasize that these results must be considered cautiously in case of a female population, due to the estrogenic effects of isoflavones.
